# Molecular Basis, Diagnostic Approaches, and Therapeutic Strategies in Colorectal Cancer—Comprehensive Review

**DOI:** 10.3390/ijms26199520

**Published:** 2025-09-29

**Authors:** Małgorzata Katarzyna Kowalska, Ahmed El-Mallul, Joanna Elżbieta Lubojańska, Weronika Hudecka, Sara Małgorzata Orłowska, Piotr Jan Lubojański, Łukasz Bednarczyk

**Affiliations:** 1 Department of Applied Chemistry, Casimir Pulaski Radom University, Street Chrobrego 27, 26-600 Radom, Poland; 2 Department of Health Sciences and Physical Culture, Casimir Pulaski Radom University, Street Chrobrego 27, 26-600 Radom, Poland; a.el-mallul@urad.edu.pl (A.E.-M.); asia.majewska13@gmail.com (J.E.L.); weronika.hudecka@gmail.com (W.H.); sara.orlowska17@gmail.com (S.M.O.); bednarczyk.lukasz21@gmail.com (Ł.B.); 3 Specialist Hospital Named Dr Tytus Chalbinski, 1 Adolfa Tochtermana Street, 26-600 Radom, Poland; piotrlubojanski@gmail.com

**Keywords:** colorectal cancer, carcinogenesis, mutations, molecular mechanisms, biomarkers, targeted therapies

## Abstract

This review covers issues related to the characteristics, diagnosis, and treatment of colorectal cancer (CRC). It discusses traditional methods of treating colorectal cancer, including surgery, chemotherapy, and radiotherapy, as well as modern approaches, including targeted therapies, immunotherapy, and innovative gene therapy strategies. Particular attention is paid to the identification of molecular subtypes of CRC, which has revolutionized treatment in advanced stages of the disease and contributed to improved patient survival. The role of biomarkers, including liquid biopsy, in diagnosis, therapy monitoring, and treatment response assessment is emphasized. The potential of artificial intelligence in planning and optimizing surgical procedures is also discussed, opening up new possibilities in personalized therapy. This article provides up-to-date knowledge on the molecular mechanisms of CRC, diagnostic prospects, and directions for the development of precision therapies, serving as a valuable source of information for both clinicians involved in the treatment of CRC and patients wishing to deepen their knowledge of the disease and modern therapeutic options.

## 1. Introduction

Cancer is one of the greatest challenges facing modern medicine. Among cancer types, colorectal cancer (CRC) ranks high in terms of incidence and mortality. CRC accounts for approximately 10% of cancer deaths worldwide and, despite numerous preventive measures, remains a significant clinical challenge. Despite significant advances in diagnosis, systemic treatment, and surgery, the mortality rate of CRC patients remains high [1,2]. For a long time, CRC treatment was based on traditional methods such as surgery, chemotherapy, and radiotherapy. However, in recent decades, thanks to the intensive development of molecular biology, knowledge and understanding of this disease has changed radically. The discovery and in-depth understanding of the molecular mechanisms leading to cancerous transformation has opened the door to the era of personalized medicine [2,3,4]. This article provides a comprehensive overview of the latest knowledge on colorectal cancer, focusing on its molecular basis. This paper also aims to raise awareness among patients and healthcare professionals, which may contribute to improving the effectiveness of therapy and clinical outcomes.

## 2. Epidemiological Data, Determinants, and Geographic and Demographic Variation in CRC

Epidemiological data play a key role in assessing the scale of the health problem that is colorectal cancer. Analysis of incidence, morbidity, mortality, and mortality rates allows for the identification of temporal trends and groups particularly vulnerable to the development of the disease. Among all cancers, colorectal cancer, which includes colon and rectal cancers, ranks alarmingly high in terms of incidence and mortality [5,6,7,8]. In 2022, the International Agency for Research on Cancer (IARC) reported nearly 20 million new cases of cancer (excluding melanoma) and 9.7 million deaths from the disease [5,8,9] Colorectal cancer is the third most commonly diagnosed cancer worldwide. In 2022, more than 1.9 million new cases were diagnosed, accounting for 9.6% of all cancer cases. It is also the second most common cause of cancer deaths (9.3%), after lung cancer, accounting for more than 900,000 deaths annually [5,9].

The prognosis for colorectal cancer is closely related to the stage of the disease at the time of diagnosis. Early detection of cancer limited to the intestinal wall and without metastases is associated with a high survival rate. The five-year survival rate in this group of patients is approximately 90% [10]. In contrast, in cases of advanced colorectal cancer with distant metastases, the five-year survival rate drops to only 13%, highlighting the critical importance of early diagnosis and effective screening programs [10].

**Figure 1:**
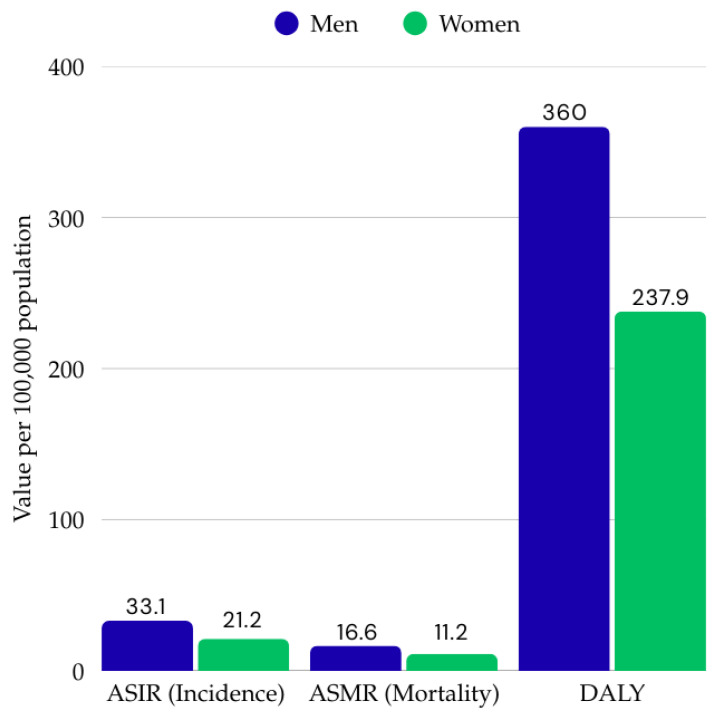
Age-standardized incidence rate (ASIR), age-standardized mortality rate (ASMR), and Disability-Adjusted Life Years (DALY) for colorectal cancer in men and women in 2019. Data derived from the Global Burden of Disease Study [11].

Gender is an important factor modifying the epidemiology of colorectal cancer. Data from the 2019 Global Burden of Disease Study show that there are significant differences between women and men in terms of incidence, mortality, and total disease burden measured by the DALY (Disability-Adjusted Life Years) index [11]. In 2019, men accounted for 57.2% of all diagnosed cases of colorectal cancer and 54.9% of deaths from the disease. The age-standardized incidence rate (ASIR) was 33.1 per 100,000 in men, compared to 21.2 per 100,000 in women [11]. The age-standardized mortality rate (ASMR) was also higher in men, at 16.6 per 100,000, compared to 11.2 per 100,000 in women (Figure 1). A similar relationship was observed for the DALY index, which reached 360.0 per 100,000 in the male population, compared to 237.9 per 100,000 in women [11]. Analysis of data from 1990–2019 confirms that there is a clear increase in the number of cases, deaths, and overall disease burden in men, indicating a continuing disadvantage in terms of the risk of developing and the effects of colorectal cancer.

In the context of the presented epidemiological data on high incidence and mortality rates due to colorectal cancer, the availability of modern methods of early detection of the disease, including population screening, becomes particularly important [6]. Colorectal cancer usually develops slowly, over many years, from precursor lesions known as adenomas [6]. This natural course of the disease creates a real opportunity for early detection of cancerous lesions and effective prevention of their progression [6,12].

**Figure 2:**
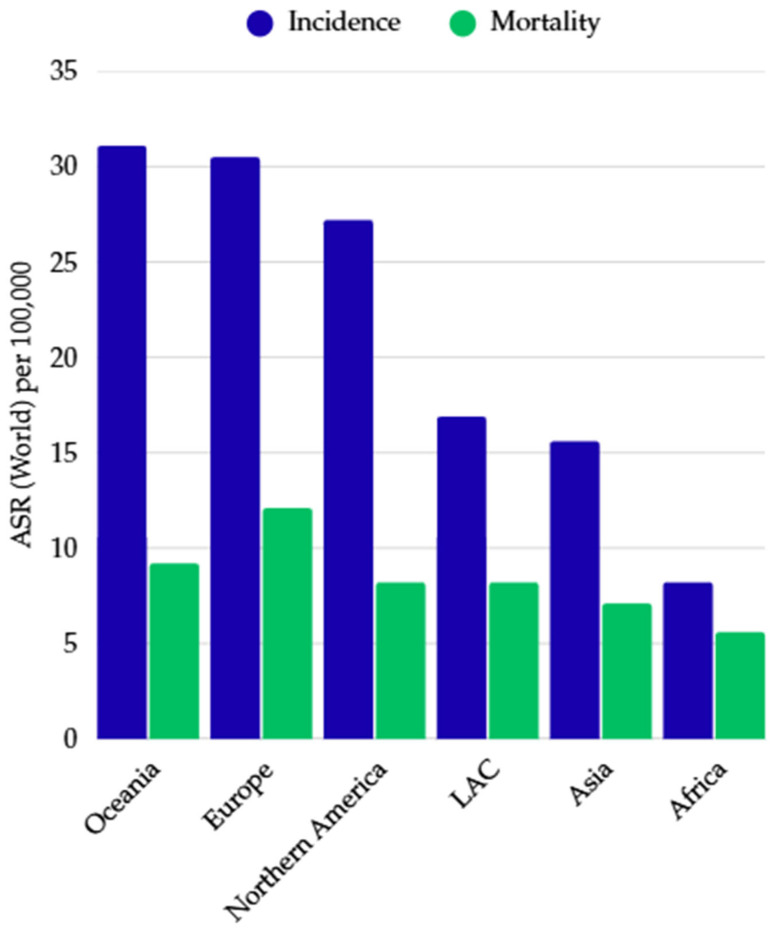
Global variation in colorectal cancer incidence and mortality—age—standardized rates (ASR, per 100,000), overall incidence and mortality (both sexes) in 2022, based on a report prepared by GLOBOCAN 2022 [9].

The incidence of colorectal cancer varies significantly depending on the geographical location and level of socio-economic development of a given region (Figure 2) [5,13]. The highest incidence rates are found primarily in highly developed countries, especially in Western Europe, North America, Australia, and New Zealand, while significantly lower rates are observed in less developed regions, including Africa and South Asia [5,13]. The variation in the incidence of the disease depending on the region of the world can be explained by differences in the prevalence of risk factors, the level of development of healthcare infrastructure, and socio-economic conditions [13]. In highly developed countries, unhealthy dietary patterns and lifestyles that promote the development of colorectal cancer play a significant role, influenced by diets rich in red and processed meat, low levels of physical activity, sedentary lifestyles, overweight, and widespread availability of various alcoholic beverages and tobacco products [5,13]. At the same time, these countries have extensive diagnostic and therapeutic infrastructure and widely implemented screening programs, which translates into better detection and reporting mechanisms and enables treatment in the early stages of the disease [13]. In addition, the increasing life expectancy in highly developed countries increases the percentage of older people, who are particularly vulnerable to developing colorectal cancer [13]. Thus, a larger number of people reaching the age of 50–60 translates into a higher overall incidence of colorectal cancer in these regions. In contrast, in developing countries, access to healthcare and screening is often limited, and the level of case reporting and epidemiological data registration is significantly lower [13]. This situation contributes to distorted recording of the actual number of cases and deaths, as well as delayed diagnosis of the disease, leading to higher mortality and poorer patient prognosis [13].

Colorectal cancer is commonly perceived as a cancer typical of the elderly, which is confirmed by epidemiological data [14]. According to a 2022 report by the International Agency for Research on Cancer (IARC), the incidence of colorectal cancer increases steadily with age. The age-standardized incidence rate (per 100,000 people) was only 1.0 in the population under 39 years of age, while it increased to 12.2 in the 60–69 age group and reached 18.4 among people over 85 years of age. However, in recent years, there has been a worrying trend of increasing incidence among younger people under the age of 50. Doctors specializing in the treatment of this type of cancer are increasingly observing an increase in the number of cases of this disease among middle-aged and younger people [14]. The fastest growth rate was observed in the 20–29 age group, averaging 7.9% per year between 2004 and 2016. This increase was slightly lower in the 30–39 age group (4.9% per year) and the 40–49 age group (1.6% per year) [14].

In addition, data from a study of 1771 patients showed that people under the age of 40 were more likely to be diagnosed at a more advanced stage of the disease [14]. Duke’s stage D occurred more than twice as often in this group than in patients aged 51–70 and more than three times as often than in people over 70 [14].

The observed phenomena may indicate the significant role of environmental factors and lifestyle in the early stages of development, such as the increase in the prevalence of obesity among children and adolescents, limited physical activity, and unfavorable changes in diet [14]. However, the high degree of disease progression at a younger age may also be a consequence of late diagnosis and previous symptomatic treatment without suspicion of cancer [14].

## 3. The Pathogenesis of Colorectal Cancer: Theories of Disease Development and Risk Determinants

The epithelium of the large intestine is characterized by high cell renewal dynamics, which promotes the development of proliferation disorders [12]. One of their consequences may be the development of polyps, which, although initially usually benign, may under certain conditions constitute a transitional stage in carcinogenesis and lead to the development of malignant lesions [12]. In the vast majority of cases (approx. 95%), colorectal cancer takes the form of adenocarcinoma [10,15,16]. The latest classification of the World Health Organization (WHO, fourth edition) distinguishes various histological variants of this cancer, including mucinous adenocarcinoma, signet ring cell carcinoma, medullary carcinoma, micropapillary carcinoma, serrated carcinoma, sieve cell carcinoma, glandular squamous cell carcinoma, spindle cell carcinoma, squamous cell carcinoma, and undifferentiated carcinoma [15,16]. Other malignant or potentially malignant tumors, such as neuroendocrine tumors (NETs), gastrointestinal stromal tumors (GISTs), sarcomas, lymphomas, and intestinal mucosal melanomas, are less commonly observed in the large intestine [16].

### 3.1. Risk Factors for Colorectal Cancer

The development of colorectal cancer is influenced by many factors, including environmental and genetic factors [10,17]. Among environmental factors—mainly related to lifestyle and eating habits—the following are particularly important: lack of physical activity; overweight and obesity; a high consumption of red and processed meat; a diet low in fiber, vegetables, and fruit; and alcohol abuse and smoking. These factors can directly affect the cells of the colon epithelium, initiating the process of malignant transformation [10,17].

Genetic factors also play an important role in the etiology of the disease, accounting for approximately 2–8% of colorectal cancer cases [17]. The most common hereditary syndromes that increase the risk of developing the disease include hereditary non-polyposis colorectal cancer (HNPCC, or Lynch syndrome) and familial adenomatous polyposis (FAP) [10,17].

Another important high-risk factor for the development of cancer is chronic inflammatory bowel disease, such as Crohn’s disease and ulcerative colitis. Persistent inflammation in turn promotes carcinogenesis and further progression of neoplastic changes, which translates into a 2–6 times higher risk of developing colorectal cancer compared to the general population [17].

### 3.2. Molecular Factors Contributing to the Development of Colorectal Cancer

Colorectal cancers are classified, among other things, on the basis of the different molecular pathways underlying their pathogenesis [15,18]. The vast majority of cases are sporadic tumors, whose development proceeds mainly through two distinct molecular mechanisms: the classic adenoma–carcinoma pathway, also known as the chromosomal instability (CIN) pathway, and the alternative microsatellite instability (MSI) pathway [15,16,17,18]. It is estimated that they account for approximately 70–85% and 12–15% of sporadic colorectal cancers, respectively [17,19]. In order to better understand these molecular pathways of neoplastic transformation, hereditary syndromes predisposing to the development of colorectal cancer, such as familial adenomatous polyposis (FAP) and Lynch syndrome (HNPCC), have been used as research models [15].

Both microsatellite instability (MSI) and chromosomal instability (CIN) describe the development of colorectal cancer as a consequence of genetic disorders leading to the inactivation of tumor suppressor genes and/or the activation of oncogenes [15]. However, this pathogenesis is not limited to changes in the sequence or structure of DNA—epigenetic mechanisms that regulate gene expression without interfering with their genetic code also play an important role, including DNA methylation, histone modifications, and chromatin remodeling [15]. Based on the analysis of hereditary syndromes predisposing to colorectal cancer, such as Lynch syndrome (HNPCC) and familial adenomatous polyposis (FAP), the epigenetic mechanisms involved in the carcinogenesis of this cancer have been better understood [15,17]. A key phenomenon is the hypermethylation of promoter regions, which leads to the epigenetic silencing of genes, including tumor suppressor genes and genes involved in DNA mismatch repair (MMR) [15]. In Lynch syndrome, there is hereditary inactivation of one allele of a DNA repair gene, while in FAP, the mutation affects one allele of the *APC* suppressor gene [17].

#### 3.2.1. Chromosomal Instability Pathway (CIN)

The chromosomal instability (CIN) pathway is a classic mechanism of colorectal carcinogenesis, referred to as the adenoma–carcinoma sequence [17,18]. Its development is based on the accumulation of mutations leading to the activation of oncogenes and the inactivation of tumor suppressor genes [17,18]. This mechanism underlies not only colorectal cancer but also many other cancers, such as pancreatic and lung cancer [20,21]. The key event initiating this pathway is a mutation in the *APC* gene, which occurs almost always in cancers developing via CIN and is also a characteristic feature of patients with familial adenomatous polyposis (FAP) (Table 1) [15]. In cancers developing along this pathway, in addition to *APC* gene mutations, disturbances in the *KRAS* and *TP53* genes are also observed at a later stage, which play an important role in the progression of cancerous changes and their transformation into malignant forms [15,18]. It is estimated that the entire process, from the development of a benign adenomatous lesion to the development of invasive colorectal cancer, takes approximately 10–15 years [18].

#### 3.2.2. Microsatellite Instability Pathway (MSI)

The microsatellite instability (MSI) pathway tends to be mutually exclusive with the chromosomal instability (CIN) pathway (CIN) [15]. As mentioned earlier, MSI is characterized by impaired function of genes responsible for repairing errors and mismatches within DNA (MMR), leading to the accumulation of mutations in microsatellite regions of the genome [15,17]. The key mechanism underlying this disorder is the epigenetic inactivation of the *MLH1* gene, most often through hypermethylation of its promoter, which results in the silencing of both MLH1 protein expression and the expression of the PMS2 protein that interacts with it [15]. In addition, *BRAF* gene mutations are often observed in tumors developing within this pathway, while *KRAS* gene mutations occur much less frequently [15].

### 3.3. Classification of Colorectal Cancer

To avoid discrepancies resulting from different classification systems for colorectal cancer, a classification based on the analysis of genetic changes has been proposed [25]. In recent years, this cancer has been further characterized based on its molecular phenotype, which includes specific biological features, gene expression patterns, and prognostic and predictive factors [25,26,27]. As a result, a molecular classification has been developed that distinguishes four consensus molecular subtypes (CMSs) of colorectal cancer: CMS1—immunological, CMS2—canonical, CMS3—metabolic, and CMS4—mesenchymal [25,26,27]. Each of the identified subtypes has a specific clinical course and different susceptibility to treatment, which means that they can serve as important prognostic and predictive factors in selecting the most appropriate therapeutic strategy [25,26].

#### 3.3.1. CMS1—Immunological Subtype (MSI)

**Figure 3:**
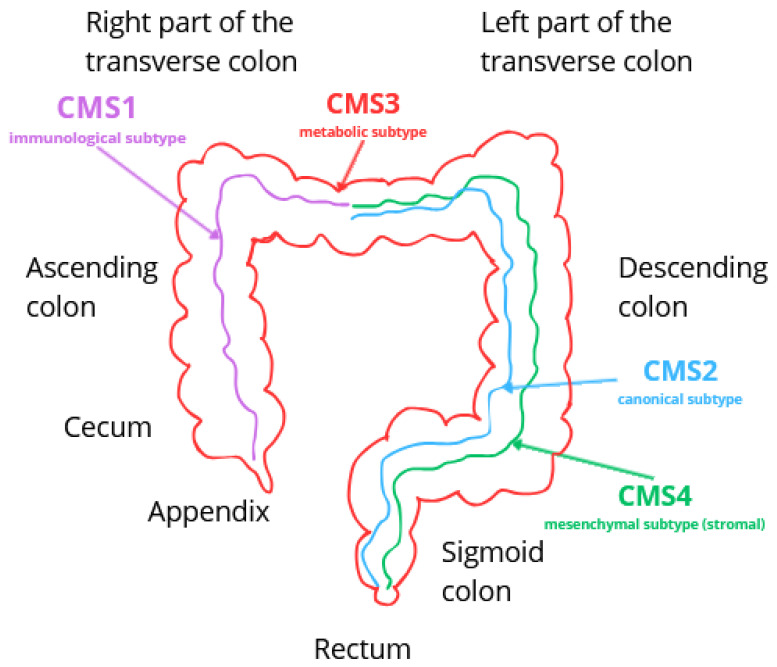
Schematic representation of the most common molecular subtype locations of CRC [25,26,27].

The immune subtype occurs in approximately 14% of colorectal cancer cases [25]. It is characterized by the presence of hypermutation, high immunogenicity, and microsatellite instability (MSI) [25,26,27]. It is distinguished by increased CpG island hypermethylation (CIMP phenotype) and a characteristic gene signature with a large number of mutations and a small number of gene copy changes. The activation of inflammatory pathways, including JAK-STAT and caspases, plays a key role in the pathogenesis [26]. The tumor environment in CMS1 is characterized by a small number of cancer-associated fibroblasts (CAFs) but a high infiltration of immune cells, which is associated with the activation of the acquired immune response [26]. The most commonly observed mutations involve the *MSH6*, *RNF43*, *ATM*, *TGFβR2*, *BRAF*, and *PTEN* genes [26]. This subtype develops mainly in the proximal part of the intestine (right side of the colon) (Figure 3), more often in women [26], and the average age of patients is around 69 years [26]. In the case of early detection of colorectal cancer, before the onset of metastases, the prognosis is relatively favorable. However, in the event of a recurrence, the prognosis becomes much more difficult. The five-year survival rate is approximately 73% [25].

#### 3.3.2. CMS2—Canonical Subtype

CMS2 is the most common subtype of colorectal cancer, accounting for approximately 37% of CRC cases [25,26,27]. The molecular hallmarks of this subtype include strong activation of the WNT/β-catenin signaling pathway and the transcription factor MYC, which regulates cell proliferation and differentiation [25,26,27]. Additional molecular alterations characteristic of CMS2 involve activation of EGFR, SRC, VEGF/VEGFR, and integrins, as well as growth factors such as TGFβ, IGF, and HER2 [26]. The canonical subtype is further characterized by low levels of DNA methylation and chromosomal instability (CIN), with a high burden of somatic copy number alterations (SCNA) but a relatively low mutation rate [26,27]. The CMS2 tumor microenvironment exhibits poor immunogenicity and contains a minimal number of cancer-associated fibroblasts [26]. Phenotypically, CMS2 demonstrates a classical epithelial profile, frequently associated with loss of APC tumor suppressor function, *KRAS* mutations, and *TP53* inactivation [26,27]. This subtype is more commonly localized in the distal colon (Figure 3), with the median patient age being approximately 66 years [26]. CMS2 is associated with a more favorable prognosis compared to CMS1, with the highest 5-year survival rates among all subtypes, a trend also observed in cases of recurrent disease [25,26].

#### 3.3.3. CMS3—Metabolic Subtype

The CMS3 subtype accounts for approximately 13% of colorectal cancer cases and is characterized by an epithelial phenotype with pronounced dysregulation of metabolic processes [26,27]. Its genomic profile includes moderate chromosomal instability (CIN), with a mutation burden lower than in CMS1 but higher than in CMS2 and CMS4 [25]. Molecular pathways implicated in its development include DNA damage repair, glutamine metabolism, and lipogenesis [26]. The tumor microenvironment is characterized by a low abundance of cancer-associated fibroblasts (CAFs) and reduced immunogenicity [26]. The most common mutations observed in this subtype are *APC*, *KRAS*, *TP53*, and *PIK3CA*, among which *KRAS* mutations occur with the highest frequency [25,26]. CMS3 tumors are distributed with similar frequency in both the proximal and distal colon (Figure 3). Clinically, CMS3 is associated with a relatively favorable prognosis, with a 5-year overall survival rate of approximately 75%, regardless of disease stage [25,26].

#### 3.3.4. CMS4—Mesenchymal Subtype (Stromal)

The mesenchymal subtype (CMS4), which accounts for approximately 23% of CRC cases, is characterized by high invasiveness and the most aggressive clinical course among all subtypes [25,26,27]. Its key molecular features include high expression of the transforming growth factor β (TGFβ) pathway, stromal invasion, activation of angiogenic processes, and an enhanced inflammatory response within the tumor microenvironment [27]. The gene signature indicates the presence of chromosomal instability (CIN), with numerous copy number alterations and a relatively low mutation burden [26]. Typical mutations associated with CMS4 include *APC*, *KRAS*, *TP53*, *PIK3CA*, *TOP1*, and *CES2.* CMS4 tumors are most often localized in the distal colon and occur somewhat more frequently in men [26] (Figure 3). The mesenchymal subtype of colorectal cancer is associated with the poorest prognosis, frequent disease recurrence, and reduced treatment efficacy [27], with an overall 5-year survival rate of approximately 62% [25].

## 4. Secondary Prevention and Diagnosis of Colorectal Cancer

### 4.1. Sreening Tests

The authors of [17] point out that when colorectal cancer is suspected in a patient, a physical examination of the abdomen, a thorough family history (degree of consanguinity, age at the time of cancer diagnosis, or type of cancer) and a review of the patient’s medical history should be undertaken. An important avenue for detecting colorectal cancer is screening, which most often involves people aged 50–75 [17,28]. A two-step screening strategy is commonly used [29]. It consists of sensitive tests based on the detection of molecular markers and blood in a stool sample [29]. Population participation in screening ranges from 16.1% to 68.2% [17]. The neoplastic transformation of adenoma to cancer is slow, so early intervention can detect adenomas by performing a colonoscopy. Removal of adenomatous polyps from the colon and rectum reduces the risk of colorectal cancer [30,31]. Screening programs lead to a reduction in colorectal cancer-related mortality and morbidity, as evidenced by fecal occult blood testing and flexible sigmoidoscopy [32].

Common screening diagnostic methods used in Western countries include fecal immunochemical test for hemoglobin (FIT), fecal occult blood test with guaiacol reagent (gFOBT), colonoscopy, sigmoidoscopy, and per rectum examination [17,28,31,32]. Each patient should be evaluated for the presence of enlarged peripheral lymph nodes, liver enlargement, and palpable abdominal tumors [17,28]. In terms of colorectal cancer screening, the 2021 U.S. Preventive Services Task Force (USPSTF) guidelines recommend screening in individuals aged 45–75 years [29,31]. A gFOBT or FIT test should be performed annually, sigmoidoscopy every 5 years, and colonoscopy every 10 years [31]. Screening guidelines differ for patients belonging to high-risk groups, such as those with a positive family history or patients with inflammatory bowel disease, genetic predisposition, acromegaly, or polyposis syndromes. In their case, it is recommended to start a screening program earlier and perform colonoscopies more frequently. For example, in patients with hereditary non-polyposis colorectal cancer (HNPCC), U.S. guidelines recommend performing colonoscopies every two years starting at age 20 and annually after age 40 [33].

An analysis of four large randomized controlled trials showed that annual or biennial gFOBT testing reduced colorectal cancer mortality by 16% [32]. For those in high-risk groups (patients with familial syndromes of familial adenomatous polyposis (FAP) or Lynch syndrome, among others), diagnostic methods are individualized [31].

The gFOBT test detects both human and dietary hemoglobin, which may lead to false positives [17,28,31]. In contrast, the FIT test assesses the amount of hemoglobin specific only to humans and is indicated in patients with low-risk symptoms [17,28,32]. According to the National Institute for Health and Care Excellence, the FIT test is recommended for patients with abnormal bowel movements or iron deficiency anemia (especially in patients over 60 years of age) [17,28]. It provides a non-invasive screening method with acceptable accuracy and high reportability [31]. Studies [17,28] prove that at 10 μg Hb/g stool, it reduces the number of colonoscopies by 75–80% in symptomatic patients. The authors of [31] indicate that performing the FIT test on a regular annual basis detected more than 80% of cancer cases within a year of testing.

### 4.2. Diagnostic Tests

Endoscopic examinations of the lower gastrointestinal tract are the basis for the diagnosis of colorectal cancer—they allow evaluation of the colon along its entire length, detection of tumors, and collection of material for examination [31]. During sigmoidoscopy, the appearance of the left side of the large intestine is assessed, with a lower patient burden compared to colonoscopy [17,28]. Randomized trials have shown that a single-stage examination using flexible sigmoidoscopy is associated with an 18–23% reduction in morbidity and a 22–31% reduction in mortality [32]. Colonoscopy is the most sensitive and specific diagnostic method in detecting colorectal cancer [17,28]. It constitutes the only screening method for high-risk populations or is used in correlation with non-invasive methods in the general population [31]. One randomized control trial showed a significant reduction in the risk of disease at 0.69 and a decrease in mortality at 0.50 [31,32]. A cohort study showed that colonoscopy combined with the performance of polypectomy reduces the incidence and mortality of colorectal cancer [32]. According to the study [32], colonoscopy reduces the risk of death from colorectal cancer by 68%.

Modern technologies such as artificial intelligence support the interpretation of endoscopic images [17,28]. Computer-aided diagnosis (CAD) systems facilitate accurate evaluation of polyps for histologic structure [17,28]. Combination strategies—combining FOBT tests with lower gastrointestinal endoscopy [31]—are of great importance for their effectiveness in detecting colorectal cancer. This increases the accuracy of cancer detection [31]. In the United States, a multi-marker fecal DNA test, which is combined with the FIT test, is used to detect early colorectal cancer. The sDNA-FIT test detects the methylated *NDRG4* gene, *BMP3*, *KRAS* mutations, and fecal hemoglobin [34]. The sDNA-FIT test showed a significant advantage in sensitivity over single FIT tests (92.3% for DNA tests, versus 73.8% for the FIT test) [34]. This indicates that compound tests are more effective but with higher costs and a lower specificity [34].

Currently, the diagnosis of colorectal cancer also uses colorectal computed tomography (CTC), which achieves a sensitivity of ≥90% for adenomas ≥10 mm. This means high efficiency in detecting larger lesions, which can improve patient selection for further investigation [32]. In contrast, magnetic resonance imaging of the colon (MRC), which does not use ionizing radiation, achieves a sensitivity of 78.4% and 75% for ≥6 mm adenomas and advanced adenomas, respectively [32]. The procedure used to capture images of the colon during passage is colonic capsule endoscopy (CCE), which involves ingestion of a capsule equipped with cameras at both ends [32]. CTC, MRC, and CCE require bowel cleansing. When significant lesions are detected during these tests, colonoscopy should be performed [32].

There are also non-invasive stool and blood tests based on the detection of DNA, RNA, or protein biomarkers that detect genetic mutations associated with the development of colorectal cancer in intestinal epithelial cells exfoliated in the stool or present in peripheral blood [31,32].

A promising alternative for patients who reject classical diagnostic methods is an assay based on methylation of the *SEPT9* gene promoter, the mSEPT9 assay [34,35]. Septins are proteins involved in cell division processes, and their abnormal methylation can lead to cell cycle disorders. The advantage of the SEPT9 test over the FIT test applies only to the symptomatic population, with studies showing a low screening efficiency of 48.2% for this test [34]. In the prospective PRESEPT study, the sensitivity of the SEPT9 test in identifying colorectal cancer was 48% However, the sensitivity in detecting precancerous lesions such as advanced adenomas was only 14.4% [35]. This indicates the need to supplement this method with other diagnostic tools [34,35].

Also analyzed in the diagnosis of colorectal cancer are galectins, proteins capable of binding beta-galactosides, which play an important role in regulating cellular processes including adhesion or apoptosis [34]. A diagnostic test based on the determination of galectin-3 levels has a sensitivity of 70% in the early stages of the disease, making it a potentially valuable adjunctive tool.

A widely used tumor marker in clinical practice, carcinoembryonic antigen (CEA) is a glycoprotein detected in patients with cancers of the gastrointestinal tract, including colorectal cancer [36]. CEA is used to detect the presence of a tumor, assess the efficacy of therapy, monitor the course of the disease, or predict the condition of patients [36].

### 4.3. Predictive Biomarkers in Therapy (Companion Diagnostics)

In addition, biomarker tests focusing on *KRAS*, *BRAF,* or *TP53* gene mutations are used in the field of colorectal cancer diagnosis, and these, along with other biomarkers, are described in Table 2 [34,36]. 

Among the diagnostic methods discussed, liquid biopsy deserves special attention. It is a diagnostic technique that enables the detection and monitoring of cancerous changes by analyzing genetic material from tumors circulating in the patient’s body fluids [38]. This method has the potential to replace the classic tumor tissue biopsy as a more accurate tool that will allow for personalized treatment [38]. In 2019, Tie et al. [39] published a study in which they attempted to answer the question of whether the analysis of circulating tumor DNA levels can provide information on the effectiveness of adjuvant chemotherapy in patients with CRC. The study involved 96 patients with stage III colorectal cancer, from whom blood samples were taken for ctDNA and CEA marker analysis 4–10 weeks after surgery and after completion of treatment. The results confirmed the prognostic significance of using ctDNA analysis after CRC surgery. Patients with a positive ctDNA result after surgery had a lower 3-year recurrence-free rate compared to patients with a negative ctDNA result, who had a rate of 76%. In addition, the study compared the reliability of estimation of recurrence using the modern method of ctDNA analysis to the CEA marker. The study showed a slightly higher recurrence risk ratio for ctDNA of 3.8 compared to CEA, which was 3.4. However, ctDNA analysis showed a higher percentage of patients with positive results, suggesting that the use of this marker may be more beneficial in qualifying patients for adjuvant chemotherapy than the use of the CEA marker [39].

CTC and ctDNA analysis enables the detection of secondary, acquired mutations and mechanisms causing resistance to treatment that arise during cancer therapy [40]. This method allows for the prediction of patient prognosis and enables continuous monitoring of treatment. Unfortunately, this method is currently very expensive, which prevents its use in many centers [38]. One of the arguments cited by Zhou in his publication [38] in favor of liquid biopsy is that it shows the heterogeneity of the tumor and allows for the monitoring of molecular changes during treatment. Liquid biopsy analyzes tumor material circulating in the blood, which comes from different parts of the body, thus providing a more complete picture of mutations and genetic variants in real time. By comparison, tissue biopsy usually collects material from a single site, which may lead to some mutations being missed and does not reflect the dynamically changing, complete genetic profile of colorectal cancer [38]. In a study conducted by Zhang in 2023 [41], CTC-DNA analysis using next-generation sequencing (NGS) was used to monitor the mechanisms of both primary and secondary resistance to trastuzumab in patients with HER-2-positive cancer. Isolated CTC-DNA allowed the authors to reflect the complex profile of genomic heterogeneity of the disease within a single patient, which is of significant prognostic importance. The results of the above study indicated that liquid biopsy allowed for the early detection of resistance to trastuzumab [41].

In addition, miRNA, exosomes, tumor-induced platelets (TEPs), and circulating tumor-derived endothelial cells (CTECs) can be used as material in liquid biopsies [42,43]. Exosomes (EVs) derived from colorectal cancer contain parts derived from genetic material, transcriptome fragments, and secretome fragments. Based on the analysis of EV content, it is possible to control proliferation, angiogenesis, metastasis, and immune response modulation [43,44]. One of the components of exosomes (exosomal dipeptidyl peptidase IV (DPP4)) has been identified as an effective diagnostic biomarker for CRC [45]. Such exosomes stimulate angiogenesis through activation of the SMAD pathway. At the same time, inhibition or silencing of DPP4 results in tumor growth arrest [45]. CTEC cells are used in LB as a diagnostic aid in the early detection of cancer and are used in antiangiogenic therapy for colorectal cancer [38]. The specific role of CTECs is to provide information on angiogenesis in cancer. There are studies that indicate that CTECs have predictive value in patients with metastatic colorectal cancer treated with bevacizumab [38]. Their presence in body fluids is conditioned by endothelial cell turnover, destruction of tumor vessels as a result of therapy, or remodeling in the progression of cancer [38].

## 5. Classic Therapeutic Strategies in Colorectal Cancer

The treatment of colorectal cancer (CRC) is primarily based on traditional oncological methods. These include surgery, chemotherapy, and radiotherapy, which are characterized by a documented efficacy and safety in patients [46]. Currently, patient treatment is based on combination therapy to increase the effectiveness of treatment and prevent recurrence of the disease. Despite the many treatment options available for CRC patients, the standard treatment remains surgery in combination with chemotherapy or radiotherapy [47]. The treatment strategy is selected individually based on the guidelines of the European Society for Medical Oncology (ESMO) (2020, 2023) [47,48], as well as other international recommendations, including those of the NCCN (USA), ASCO (American Society of Clinical Oncology), and the Japanese Society for Cancer of the Colon and Rectum (JSCCR). The decision takes into account the stage of advancement, histopathological type, molecular profile of the tumor, and its location [49,50,51].

The primary method of treating stage I-III colorectal cancer is surgical treatment. The aim of the surgery is to remove the tumor and surrounding lymph nodes. In high-risk stage II patients and stage III CRC patients, adjuvant FOLFOX or CAPOX chemotherapy is recommended for a period of 3–6 months [48]. FOLFOX therapy is based on the administration of folic acid, 5-fluorouracil, and oxaliplatin, while CAPOX chemotherapy consists of capecitabine and oxaliplatin [48]. The ESMO, NCCN, and JSCCR guidelines consistently point to the benefits of adjuvant chemotherapy, although they differ in details regarding duration and choice of regimen [49,50,51,52]. In 2018, IDEA (International Duration Evaluation of Adjuvant Therapy) showed no differences in the group of patients with T1-3N1 CRC who received adjuvant chemotherapy for 3 or 6 months. A difference in disease-free survival was observed in patients with stage T4 and/or N2 who received 6 months of chemotherapy compared to patients who received only 3 months of chemotherapy [49]. In addition, according to the JRCCR, adjuvant chemotherapy should be started within 4–8 weeks after CRC resection [50]. The ESMO recommends that adjuvant therapy should be started as early as possible, no later than 8 weeks after surgery [47]. An analysis of the SCOT randomized clinical trial conducted in 2024 showed that adjuvant therapy started 6 weeks after surgical treatment was associated with a lower percentage of disease-free survival [53].

Cancer of the terminal segment of the large intestine, the rectum, differs in terms of recommendations compared to colon cancer. The standard treatment for rectal cancer is radiotherapy combined with chemotherapy prior to surgery (chemotherapy with 5-FU or capecitabine combined with radiotherapy). The use of preoperative chemotherapy reduces the size of the tumor and increases the likelihood of radical resection after surgery [54]. American guidelines (NCCN, ASCO) highlight the effectiveness of TNT therapy in rectal cancer. Total neoadjuvant therapy (TNT) is a comprehensive approach that includes preoperative treatment, chemoradiotherapy, and early chemotherapy. This strategy improves the rate of complete tumor regression and may facilitate the implementation of a “watch and wait” strategy in selected patients. The W&W strategy applies to patients who have achieved clinical remission confirmed by endoscopic and MRI examinations during neoadjuvant treatment and who do not present any clinical symptoms. In such patients, surgical intervention is avoided, and the patient is placed under intensive observation. This method can be used in specialized centers, which enable close monitoring of patients by performing the above-mentioned tests every 3–6 months [55,56].

With regard to stage IV colorectal cancer, its metastatic form and treatment guidelines are more varied and depend on factors such as location, site and number of metastases, and the patient’s general condition. For colorectal cancer with limited metastases to the liver or lungs, the NCCN recommends primary use of chemotherapy with targeted therapy, followed by surgical removal of metastases [51]. In cases of significantly disseminated disease, palliative chemotherapy with targeted therapy is used to prolong and improve the patient’s quality of life, often in combination with molecularly targeted therapies [48,51]. According to the ESMO, NCCN, and JSCCR, patients with unresectable metastatic CRC should be evaluated every 2–3 months to reassess their eligibility for surgical treatment [48,51,52].

The above summary of guidelines (ESMO, NCCN, ASCO, JSCCR) emphasizes the significant role of an individualized approach to patients with colorectal cancer and multimodal therapy. American and European guidelines emphasize the importance of molecular testing, which determines the selection of systemic treatment and the possibility of using targeted therapy or immunotherapy [47,48,51].

## 6. Modern Methods of Treatment of Colorectal Cancer

### 6.1. Molecularly Targeted Therapies and Immunotherapy in Colorectal Cancer

Targeted therapy is a type of cancer treatment that relies on acting on specific biological molecules to stop the growth and proliferation of cancer cells [57,58]. Stratification of patients with metastatic colorectal cancer based on biomarkers enables the use of an individually tailored targeted therapy that corresponds to the mutational profile of the tumor [59,60]. Targeted therapy is based on the use of small molecules that can easily penetrate cells and monoclonal antibodies [57]. The advent of targeted therapies has made possible precise interventions that target molecular pathways crucial to tumor progression [61]. According to recent studies, the use of targeted therapies in patients with metastatic colorectal cancer extends overall survival time and also improves the patient’s quality of life, as confirmed by studies of cetuximab, panitumumab, and anti-VEGF drugs [62,63].

In addition, the use of targeted therapy in patients with metastatic colorectal cancer has extended overall survival to about 3 years [59]. Therapeutic options for the treatment of colorectal cancer have expanded to include biologic drugs including monoclonal antibodies directed against the epidermal growth factor receptor (EGFR) and anti-angiogenic drugs that block the VEGF signaling pathway [30,59]. Despite significant advances in therapeutic strategies, current treatments, which include chemotherapy and targeted therapies, face significant limitations in terms of systemic toxicity, development of resistance, or lack of specificity against the tumor’s mutational profile [61].

#### 6.1.1. Therapies Involving Anti-VEGF and Anti-EGFR Antibodies and Targeting the VEGF-VEGFR Signaling Pathway

The introduction of targeted therapies for the treatment of colorectal cancer has contributed to significant advances in improving treatment outcomes and prolonging patient survival [35].

**Figure 4:**
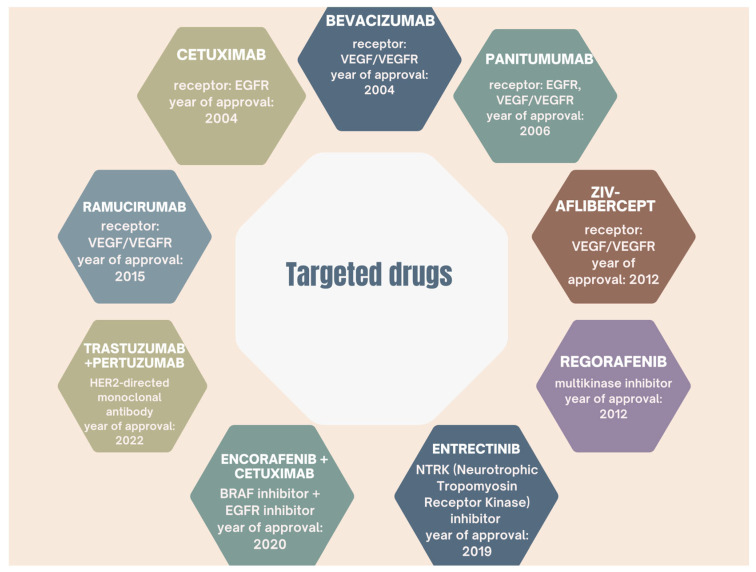
Summary of selected and FDA-approved targeted therapies for the treatment of colorectal cancer—molecular targets and year of approval [30,35].

The U.S. Food and Drug Administration (FDA) has approved a number of targeted drugs against specific checkpoints, including vascular endothelial growth factor (VEGF) and its receptors (VEGFR) [30]. VEGF plays a key role as an angiogenic factor in both primary and metastatic colorectal cancer [61]. The presence of this marker can be found early in tumors’ development, and it is associated with increased angiogenesis and more microvessels in colon tumors [61]. Other targeted drugs approved by the FDA are shown in Figure 4.

##### Anti-VEGF Therapies

Bevacizumab is a monoclonal antibody targeting VEGF-A, used in combination with chemotherapy to treat metastatic colorectal cancer regardless of RAS or BRAF mutation status [60,61]. It leads to blocking the interaction of VEGF-A with its receptor VEGFR, leading to inhibition of signaling pathways responsible for vessels’ formation [61]. Anti-VEGF therapy leads to normalization of tumor vascularization, which improves the process of drug delivery to tumor cells [60]. The process of angiogenesis is one of the most important processes that accelerate the progression of colorectal cancer [60]. Angiogenesis is the formation of new blood vessels, which are formed from endothelial progenitor cells [61]. As reported by the authors of [63], the AVF2107 study showed that the addition of bevacizumab to chemotherapy consisting of irinotecan, 5-fluorouracil, and leucovorin significantly improved overall survival (mOS 20.3 months) and progression-free survival at 10.6 months compared to placebo.

Aflibercept is a recombinant fusion protein that binds VEGFA, VEGFB, and placental growth factor, which, when combined with chemotherapy in the form of 5 fluorouracil, leucovorin, and irinotecan (FOLFIRI), increases the ORR by 8.7% and improves median disease progression-free survival by 2.2 months compared to the FOLFIRI chemotherapy regimen alone [35,61]. This therapy is associated with a high incidence of side effects, such as hypertension, thromboembolic complications, diarrhea, and neutropenia [57].

Ramucirumab binds directly to the extracellular domain of VEGFR-2, blocking the attachment of all VEGF ligands, leading to inhibition of angiogenesis and tumor progression [61]. In the RAISE trial, ramucirumab combined with the FOLFIRI chemotherapy regimen showed improved median disease progression-free survival (5.7 months vs. 4.5 months) and overall survival (13.3 months vs. 11.7 months) compared to placebo with FOLFIRI [63]. The most common side effects include neutropenia, hypertension, diarrhea, and fatigue [57].

A new oral tyrosine kinase inhibitor is fruquitinib, which was approved in 2023 for treatment III based on the results of the FRESCO-2 trial [57]. It blocks signaling through VEGRF1/2/3 receptors, and the authors of [35] report that the use of fruvitinib in refractory patients led to a 1.5% improvement in ORR, a 1.9-month increase in median progression-free survival, and a 2.6-month increase in mOS compared to the placebo group [35].

The authors of [63] note that the efficacy of anti-VEGF therapy may be limited by tumor characteristics such as genetic alterations or vascular mimicry. Resistance to anti-VEGF therapy is also due to elevated levels of angiopoietin-2, which leads to reduced efficacy of bevacizumab [61]. Therefore, research into effective targeted therapies is still needed to counter both resistance and tumor progression.

##### Anti-EGFR Therapies

Therapy using humanized monoclonal antibodies, such as cetuximab, works by selectively recognizing the epidermal growth factor receptor. The first approved targeted drug was cetuximab in 2004 [30]. In the case of the epidermal growth factor receptor, it is its activation that triggers a signaling cascade that promotes proliferation, angiogenesis, migration, and cell adhesion. Hence, deregulation of this pathway leads to tumor transformation by generating resistance to apoptosis and causes metastasis [61].

Importantly, the efficacy of anti-EGFR therapy may also depend on the location of the tumor. A meta-analysis of five clinical trials showed that the efficacy of therapy depends on the location of the primary tumor. Epidermal growth factor receptor inhibitors showed the greatest efficacy in treating tumors located on the left side of the colon, while bevacizumab had greater efficacy in right-sided metastatic colorectal cancer [35]. Anti-EGFR antibodies (cetuximab, panitumumab) require a RAS and BRAF wild-type status to be effective [63]. According to the FIRE-3 clinical trial, the objective response rate (ORR) was 66% for cetuximab and the median overall survival (mOS) was 31.1 months, with an ORR of 59% for bevacizumab and a mOS of 25.6 months [35]. The PEAK trial evaluated treatment outcomes in colorectal cancer patients with *RAS* and *BRAF* mutations who received chemotherapy consisting of 5-fluorouracil, leucovorin, or oxaliplatin in combination with panitumumab or bevacizumab targeted therapy. The objective response rate and median overall survival differed slightly in favor of the group that received panitumumab [35].

Cetuximab is a chimeric IgG1 antibody that blocks the extracellular domain of EGFR, preventing the binding of natural ligands and thereby inhibiting the transmission of proliferative and migratory signals [57]. It inhibits tumor growth, especially in patients with mutations in the *RAS* gene. A new therapeutic approach is combining cetuximab with immunotherapy, which offers new treatment options for patients refractory to standard therapy [61]. In the CRYSTAL III trial, combining cetuximab with FOLFIRI chemotherapy reduced the risk of disease progression by 15% compared to chemotherapy alone [57].

The authors of [61] also report panitumumab as a human monoclonal antibody that binds to EGFR, blocking pathways that promote cell proliferation and tumor growth. The PRIME trial showed that combining panitumumab with the FOLFOX chemotherapy regimen (leucovorin, 5-fluorouracil, oxaliplatin) in patients with *KRAS* gene mutation led to a prolongation of median time free of disease progression (9.6 months vs. 8 months) [57]. Clinical trials of other anti-EGFR antibodies such as necytizumab and nimotuzumab are still ongoing, but their efficacy in the treatment of colorectal cancer, especially metastatic colorectal cancer, needs further clinical validation [63].

The ASPECCT trial demonstrated that overall survival and quality of life of patients were comparable after both panitumumab and cetuximab [62]. The authors of [62] emphasize that the use of panitumumab was associated with fewer infusion reactions but with a higher incidence of hypomagnesemia. In terms of skin lesions such as rashes or other side effects, the frequency was similar for both drugs [62].

#### 6.1.2. BRAF Kinase-Targeted Therapy

Mutations of the *BRAF* gene occur in about 8.5% of patients with colorectal cancer [60]. Class I mutations enable signal activation independent of *RAS* pathway activation, acting as monomers. Class II mutations act as dimers and are also independent of *EGFR* and *RAS*. In contrast, class III mutations are more dependent on EGFR activity and may be sensitive to treatment with EGFR inhibitors [60]. Studies have shown that the use of anti-EGFR therapy has proved ineffective in patients with *BRAF-V600E* mutations. A therapeutic breakthrough appeared to be the introduction of a *BRAF* inhibitor in the form of encorafenib combined with an anti-EGFR antibody [60]. As of 2020, the standard of care for second-line therapy for colorectal cancer is the combination of the anti-EGFR antibody cetuximab with the oral *BRAFV600E* inhibitor [35]. The BEACON trial showed that with the combination of encorafenib and cetuximab, there was an improvement in disease progression-free time and overall survival compared to standard therapeutic options corresponding to second- and third-line treatment [62]. This combination increased the response rate to 19.5% compared to 1.8% in the control group using chemotherapy and cetuximab [60]. The results showed that both triple and double therapy provided a significantly longer overall survival of 9.3 months [57]. Based on these data, the American Society of Clinical Oncology recommends the use of the combination of encorafenib and cetuximab in patients with *BRAF-V600E* mCRC who have failed to respond to at least one prior line of therapy [57].

The treatment of metastatic colorectal cancer also includes regorafenib, which is an oral multikinase inhibitor that blocks *BRAF* or VEGFR1/2/3 signaling, leading to inhibition of tumor angiogenesis [57,61]. As reported by the authors of [35], regorafenib prolonged survival in previously treated patients with metastatic colorectal cancer. It showed a 0.6% improvement in ORR, extending mOS by 1.4 months and median progression-free survival by 0.2 months [35]. In addition, regorafenib also exhibits antiproliferative and anti-tumor effects by affecting pathways related to metastasis and immune immunity [61].

#### 6.1.3. PD-1/PD-L1 Checkpoint Inhibitors in the Treatment of Colorectal Cancer

In addition, immunotherapy is a promising therapeutic method for the treatment of colorectal cancer [30]. The following techniques are used in the field of immunotherapy: monoclonal antibody therapy or administration of immune checkpoint inhibitors [30]. Of particular interest is the interaction of the programmed cell death 1 (PD-1) receptor present on T cells with the PD-L1 ligand, which is produced by tumor cells [35]. Binding of the receptor to the ligand enables cancer cells to evade the immune response by inhibiting T-cell activity. Blocking this interaction allows T lymphocytes to reactivate and enhances immune elimination of the tumor by increasing the activity of the immune system [35,57]. Immunotherapy with anti-PD-1 antibodies has been approved as a first-line treatment for metastatic colorectal cancer with impaired unpaired base repair mechanisms or high microsatellite instability [60]. There are five FDA-approved PD-1 inhibitors, three of which are approved for the treatment of colorectal cancer [63].

Pembrolizumab was approved by the FDA in 2020 for patients with inoperable or metastatic colorectal cancer with high microsatellite instability or unpaired base repair deficits who were not receiving systemic therapy [57]. For colorectal cancer with microsatellite instability, pembrolizumab (a monoclonal antibody against PD-1) showed higher efficacy than standard chemotherapy combined with bevacizumab or cetuximab [35]. An ORR of 45.1% was achieved. The most common side effects of pembrolizumab include diarrhea, fatigue, and nausea [57].

Currently, pembrolizumab is an approved drug for first-line treatment, while nivolumab or its combination with ipilimumab is indicated for second-line treatment after prior chemotherapy [35].

Nivolumab is a humanized monoclonal antibody of the IgG4 class that was approved for the treatment of patients with colorectal cancer in 2017 [57]. Nivolumab provides durable responses to treatment (at 12 months), with an overall survival rate of 69% in patients with metastatic colorectal cancer with a defect in the unpaired base repair mechanism [63]. Nivolumab is a safe drug, with the most common side effects being rash and pruritus [57]. Serious side effects are rare, in the form of ventricular arrhythmias, neuropathy, or iritis [57].

According to statistics [35], up to 95% of metastatic colorectal cancer cases are tumors with stable microsatellites, in which immunotherapy monotherapy has a minimal therapeutic effect. Currently, combination therapy regimens are being developed: among others, the combination of immunotherapy with tyrosine kinase inhibitors. In clinical trials, the combination of pembrolizumab with lenvitinib resulted in an ORR of 22% and an mOS of 7.5 months for patients previously treated with chemotherapy [35].

Dostarlimab was approved by the FDA in 2023 for the treatment of locally advanced rectal cancer with high microsatellite instability or unpaired base repair deficits [57]. The study confirms that the use of dostarlimab in monotherapy led to a complete clinical response, with no relapse or progression at six-month follow-up. The treatment was well tolerated, and no serious side effects were observed [57].

#### 6.1.4. Advances and New Strategies for Targeted Therapy

A rare mutation in colorectal cancer is amplification or overexpression of *HER2* [35]. It is estimated that about 5% of metastatic colorectal cancer cases have an *HER2* mutation [57]. In 2023, therapies for metastatic colorectal cancer in the form of trastuzumab and tucatinib were approved for HER2+ patients and *RAS* mutation [35]. There are now several standard treatment options for patients with this disease subtype, including combinations of trastuzumab with pertuzumab, tucatinib, and trastuzumab deruxotecan [62]. The MOUNTAINEER trial showed promising results with the combination of trastuzumab and tucatinib, with an objective response rate of 38.2% and a median duration of response of 12.4 months [57].

Turning now to less common tyrosine kinase gene fusions such as *RET* or *NTRK*, in 2024 the US Food and Drug Administration approved repotrectinib for the treatment of patients with *NTRK* fusions. Selpercatinib (a selective RET kinase inhibitor) was approved in 2022 for the treatment of patients with metastatic colorectal cancer. It showed an objective response rate of 20–30% and a median duration of response of 9.4 months [35].

Signaling pathways such as Wnt/β-catenin and TGF-β have significant therapeutic potential for colorectal cancer [61]. Mutations in TGF-β receptors and SMAD proteins are associated with an aggressive tumor phenotype [61]. Drugs that directly target the TGF-β pathway are in clinical trials [61]. In contrast, overactivation of the Wnt/β-catenin pathway occurs in more than 90% of patients with colorectal cancer. Increased levels of β-catenin are associated with a poorer prognosis and a more aggressive disease course.

For *KRAS*-mutated cancers characterized by resistance to EGFR inhibitors, the oral *KRAS* inhibitors sotorasib and adagrasib are applicable. These drugs have been included in the National Comprehensive Cancer Network guidelines in combination with cetuximab or panitumumab as a treatment for patients with refractory colorectal cancer with the *KRAS G12C* mutation [62].

### 6.2. Other Systemic Treatments for Colorectal Cancer

Gene therapy for the treatment of colorectal cancer involves modifying or correcting defective genes and preventing overexpression of certain genes. Colorectal cancer can be caused by either point mutations, oncogenes, disruption of protooncogenes, or loss of activity of suppressor genes [30]. Genetic defects are thought to account for at least 30% of colon cancer cases. The main benefit of gene therapy is the ability to transfer specific genes to selected cells, in this case altered tumor cells, thus inhibiting the abnormal function of the mutated gene and slowing tumor growth [30].

Another method used to treat colorectal cancer is adoptive T-lymphocyte therapy, which enhances anti-tumor immunity. It involves adding chimeric antigen receptors (CAR-T) to effector T cells [30]. T lymphocytes are harvested from the patient or from a tissue-compatibility antigen-compatible donor (allogeneic), then cultured ex vivo and modified by CAR receptors’ insertion. The main targets of this therapy are carcinoembryonic antigens (CEAsw), guanylyl cyclase, or TAG-72 glycoprotein [63]. Studies are underway evaluating the effect of CAR-T lymphocytes in the treatment of colorectal cancer’s metastasis to the liver [63]. A limitation of CAR-T cells is that they have a limited ability to infiltrate solid tumors due to their structure [63].

There are studies that evaluate the efficacy of complement system inhibitors in the immunological control of colorectal cancer. An animal model included mice with colorectal cancer treated with complement system inhibitors—cobra venom, humanized cobra venom, recombinant *Staphylococcus aureus* superantigen proteins [30]. The authors [30] emphasize that the treatment method of inhibiting the complement system resulted in slowing tumor growth, increasing the immune response, or reducing the effect of the unfavorable tumor microenvironment.

In conclusion, significant progress has been made in recent years in improving the outcome of colorectal cancer treatment, in terms of chemotherapy, targeted therapy, and surgical approaches. Trying to overcome treatment resistance, minimizing systemic toxicity, and improving the efficacy of long-term therapy remain major challenges [61].

## 7. Contemporary Surgical Techniques—Innovative Approaches in Surgery

The authors of [30] point out that in typical cases of colorectal cancer, surgical removal of the tumor in its entirety is necessary. However, nearly 25% of patients with colorectal cancer are diagnosed at an advanced stage, and 20% of the remaining patients develop metachronous metastases—in such cases, the use of radical surgery alone is not sufficient [30].

In recent decades, the rapid development of medical technology has led to the development of a number of modern surgical techniques that increase the precision of surgery, shorten recovery time, and reduce the risk of complications [64]. Laparoscopic or robotic surgery has become a key development [64].

### 7.1. Robotic Surgery

The first reports on the use of robotics in colectomy date back to 2002 and are from the United States [65]. The use of robotic surgery improves the precision of the procedures, making it possible, among other things, to remove lymph nodes more accurately and to perform complex intestinal anastomoses inside the abdominal cavity [57]. Robotic-assisted surgery has resulted in better lymph node resections and significantly lower lymph leakage rates, indicating better tumor outcomes and fewer surgical complications [64]. In addition, robotic surgery enables precise dissection in narrow and complex pelvic lateral spaces [64]. This is facilitated by a stable surgical platform and enhanced 3D visualization. Robotic technology enables procedures in anatomically challenging areas such as the autonomic nerve area, among others. with greater precision than traditional laparoscopy [64]. In addition, the main advantages of robotic surgery include the elimination of the surgeon’s hand tremors, stereoscopic vision, the possibility of precise movements, and better dexterity [65]. As indicated by the conclusions of cohort studies, robotic surgery can improve the length of survival of patients with stage I to III colon cancer [65]. According to a study published in the *British Journal of Surgery*, it appears that patients undergoing robotic-assisted procedures demonstrated lower C-reactive protein levels on the first postoperative day compared with those treated by conventional methods [64]. In addition, patients operated on by robotic surgery methods experienced less bloating and returned to normal bowel function more quickly [65]. The use of robotic surgery was associated with lower levels of postoperative pain, resulting in less need for opioid medication, faster mobilization of patients, and shorter hospitalization [64]. The authors of [57,65] note the longer operative time, higher costs, and the need for operator training when using robotic surgery.

A particularly important indication for robotic surgery is rectal cancer located in the middle and lower rectum. Meta-analyses suggest that the use of a robotic system allows for a better therapeutic effect, with comparable oncological outcomes to laparoscopy [57,65]. Robotic precision and the ability to maneuver instruments into hard-to-reach pelvic spaces allows for more effective protection of neural structures [64]. In addition, the use of robotics reduces the occurrence of postoperative sexual and urinary dysfunction, as this surgical technique has been shown to be superior in protecting pelvic autonomic nerves [57,65].

### 7.2. Innovations in Surgical Planning with Intraoperative Navigation

**Figure 5:**
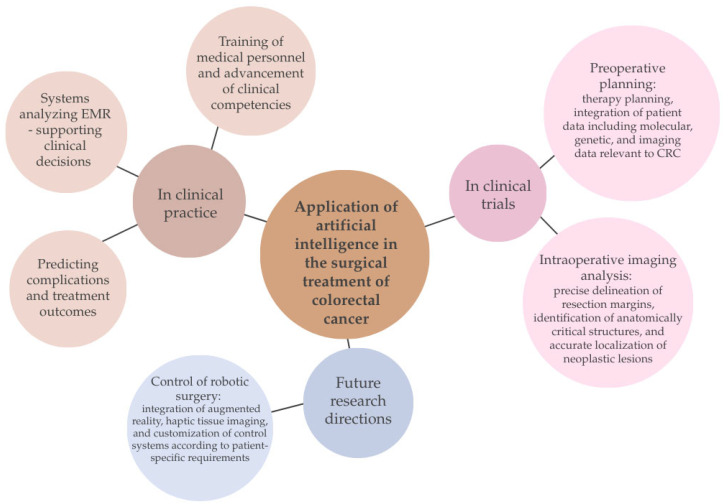
Potential areas of application for AI in the surgical treatment of patients with CRC [68,71,72,73,74,75].

Modern surgery is a rapidly developing field thanks to the combination of information technology, robotics, modern imaging techniques, artificial intelligence, and tools that allow surgeons to plan the course of an operation [66]. Modern surgical methods aim to minimize the risk to the patient by reducing postoperative complications, while at the same time striving to increase the effectiveness of the procedures performed (Figure 5). The use of intraoperative navigation is attracting increasing interest in the medical community. Thanks to the use of advanced intraoperative imaging technologies, it has become possible to precisely locate tumor lesions, mark resection margins, and identify critical anatomical structures (Figure 5) [67,68,69]. Surgical navigation can also be used to take into account surgical plans, and then the surgeon can transfer the planned surgical goal to the actual configuration of the patient [70]. A study conducted by Kok et al. between 2016 and 2019 [68] at the Netherlands Cancer Institute evaluated the use of an electromagnetic navigation system during surgery to remove locally primary or recurrent rectal cancer. The authors demonstrated that the use of the navigation system is not associated with any additional risk of complications, which is an important issue in the context of patient safety. The results of the study also indicate that the use of navigation during surgery was associated with an increase in the percentage of patients with complete resection, measured by the number of resection margins without the presence of tumor. In patients with recurrent cancer, the R0 rate was 78.9% in the group that used navigation during surgery and 48.8% in patients who were included in the cohort that did not use intraoperative navigation [68].

Modern intraoperative imaging techniques can become a valuable tool supporting surgeons during procedures to minimize the risk of intraoperative organ damage. Available data confirm that it is possible to avoid approximately 35% of complications after surgical procedures by using modern methods and appropriate interventions [71]. In 2023 Kitaguchi et al. presented important data in the context of the future use of automatic organ recognition models [71]. The tool developed by the authors to recognize the ureter and other anatomical structures during laparoscopic colon surgery has increased the speed and precision of surgeons’ actions. The results of the study indicate that the use of innovative intraoperative navigation methods, such as an image navigation system using AI-based CV (computer vision) technology, can partially compensate for the lack of practical experience, especially in less experienced operators [71].

### 7.3. Laparoscopic Surgery and Its Advantages over Traditional Surgery

A breakthrough in oncological surgery was the use of laparoscopic surgery in colon resection, which gained popularity in the 1990s. Currently, it is estimated that 50% of colon resections are performed using laparoscopic surgery [76]. In recent years, there has been a dynamic development and popularization of robotic surgery, which is supported by greater effectiveness of treatment and patient safety. Despite the growing popularity of robotic surgery, the results of some studies suggest that laparoscopic techniques are equivalent in terms of treatments’ effectiveness [76]. In 2024, Negrut [76] published a systematic review of existing studies, the aim of which was to compare the laparoscopic method with robotic surgery, taking into account the duration of the operation and total hospitalization, conversion rates, anastomotic leakage rates, and the total number of lymph nodes removed. A total of 50,771 patients were included in the study, with 21.75% undergoing robotic surgery and 78.25% undergoing laparoscopic surgery. The results of the pooled studies indicate that there was no difference between the two methods of colorectal cancer treatment in terms of anastomotic leakage. However, it was observed that patients who underwent robotic surgery had shorter hospital stays and a greater number of lymph nodes removed. On the other hand, laparoscopic procedures were associated with shorter total operating times compared to robotic resections. Differences were also observed in the conversion rate to traditional surgery, which was higher for laparoscopic procedures. The decision to choose a particular surgical technique was probably made by the surgeon in order to ensure patient safety and reduce adverse effects. From the perspective of the discussed assessment, this should not be taken as evidence of lower quality of laparoscopic surgery compared to robotic surgery, as the choice of surgical method depends on the individual assessment and experience of the surgeon [76].

The laparoscopic surgery method is distinguished by the fact that it is a minimally invasive method which, with the use of specialized tools and a camera, allows for the resection of colon cancer. Compared to the traditional method, it reduces blood loss, lowers the risk of postoperative wound infection due to a smaller incision in the abdominal wall, and shortens the hospital stay, resulting in faster recovery of patients after surgery [77]. Both techniques have their advantages and limitations, and their selection depends on many factors, including the stage of the disease, the patient’s general condition, and the experience of the surgical team [77]. In 2021, Schootman et al. [78] conducted a study in which they attempted to assess whether the treatment outcomes of patients with locally advanced colorectal cancer (T4) who underwent laparoscopic colectomy (LC) and open colectomy (OC) were comparable. The aim of the analysis was to determine whether the laparoscopic method could be a safe and effective alternative to open surgery in all patients and in more advanced cases of the disease. Patients with non-metastatic colorectal cancer, invading surrounding organs/structures or penetrating the visceral peritoneum, who underwent LC or OC were eligible for the study. Patients were evaluated 30 days after surgery, analyzing, among other things, the duration of surgery, anastomotic leakage, mortality, the need for reoperation, length of hospital stay, risk of wound infection, postoperative intestinal obstruction, and other cardiovascular complications. Based on the analysis of the patients studied, it can be concluded that laparoscopic surgery is an equally effective method of treating locally advanced colorectal cancer in both obese patients and those with difficult tumor locations. However, the 2-day shorter hospital stay associated with laparoscopic treatment may contribute to a reduced risk of cardiovascular complications, which is an important argument in favor of the laparoscopic method [78].

### 7.4. The Use of AI in the Surgical Treatment of Colorectal Cancer

The development of AI-based technology in the surgical treatment of colorectal cancer has contributed to a significant improvement in therapeutic outcomes. It has increased the percentage of radical resections, reduced the number of postoperative complications, improved long-term patient prognosis, and reduced the number of disease recurrences [67].

The influx of numerous studies since 2020 on the use of AI in colorectal cancer therapy testifies to the dynamic development of this new technology. The authors have identified areas in which the use of AI can break with traditional medicine, revolutionizing modern surgical methods, which will translate into maximizing the effectiveness of existing treatments [54]. Contemporary research into the use of artificial intelligence in colorectal cancer surgery focuses on supporting surgeons in planning and intraoperative navigation, the importance of which has been described above. In addition, AI is used to predict complications and treatment outcomes, as well as in education and the training of practical skills (Figure 5) [54]. Some of the models developed are already in clinical use, others remain at the research stage, and still others are setting the future direction for the development of artificial intelligence. In 2025, Dedhania et al. [74] determined the potential of an AI-based predictive model called C the Signs, introduced into clinical practice, in a group of 894,275 patients. The aim of the developed system was to determine the risk of CRC based on the patient’s electronic medical records [EMRs]. The results of the study showed a sensitivity of 93.8% and a specificity of 19.7% for the method used to identify patients at risk [74]. The machine learning used in the above-mentioned system is one of the branches of AI used in medicine, and its task is to create algorithms based on available data that can be used to determine the risk of disease, plan treatment, predict outcomes, anticipate complications, or assess the chance of cancer’s recurrence. Another study confirming the effective use of AI in preoperative planning is the study conducted in 2020 by Li et al. [72]. The authors developed artificial intelligence-based models that predict peripheral nerve invasion (PNI) and *KRAS* mutations in patients with colorectal cancer. The results of the study indicate that the use of an AI-supported model can be a valuable tool for determining the risk of postoperative complications and the presence of significant genetic mutations and matching the most effective therapy depending on the stage of the disease and the characteristics of the tumor [72]. In this study, artificial intelligence based on computed tomography was used to detect *KRAS* mutations and the presence of PNI in patients with colorectal cancer, which were unfavorable prognostic factors. Preoperative detection of PNI allowed for the prediction of a high chance of lymph node metastasis in these patients and the planning of chemotherapy treatment after colorectal cancer surgery [72]. Further evidence supporting the effectiveness of AI models in surgical practice comes from a study conducted by Su et al. in 2023 [73] on the prediction of benign anastomotic strictures (BASs) after rectal cancer resection [73]. Based on data collected from 1973 patients who underwent rectal resection, it was shown that the system accurately predicted the occurrence of BASs, improving the quality of life of these patients after surgery. Furthermore, when analyzing the data, the model indicated that potential factors increasing the risk of stricture are prophylactic ileostomy, duration of surgery, and anastomotic leakage. The effectiveness of AI is supported by the results obtained by one of the systems, which achieved an AUC = 0.888, sensitivity = 84.6%, and specificity = 79.1% in the assessment of patients who are at risk of anastomotic stricture after surgery. The use of models assessing the risk of complications will allow preventive measures to be implemented to increase the effectiveness of diseases’ treatment and patient comfort [73]. The studies cited above indicate that many centers use artificial intelligence as a support tool. Although a significant proportion of AI-based models are currently still in the research phase, their primary purpose is to increase diagnostic accuracy and support the decision-making process of clinicians.

The integration of AI with robotic surgery increases precision, supports surgeons’ decision-making, and improves patient safety in real time during oncological procedures using methods such as 3D modeling, augmented reality (AR), image-free segmentation, tactile imaging, and adaptive control frameworks (Figure 5) [67]. The implementation of artificial intelligence in robotic surgery has evolved over the years. Initially, AI allowed for the automation of basic surgical procedures, which was intended to reduce the physical burden on the surgeon. Currently, its role is much broader, with modern AI-based systems enabling the planning of the course of surgery, analyzing the surgical field during the procedure, and providing the surgeon with information about any adverse events that may occur. Artificial intelligence, which has been gaining popularity in recent years, brings many benefits to oncological surgery, although its full potential is still the subject of much research. However, despite promising results, the implementation of AI-assisted robotic surgery is limited by high costs and the limited availability of the technology in many oncology centers [79].

## 8. Conclusions

Colorectal cancer remains one of the most serious cancers, characterized by a complex, multifactorial etiology and diverse clinical course. Advances in understanding the molecular mechanisms of carcinogenesis, including suppressor gene mutations, oncogene activation, and DNA repair system disorders, are key to improving both diagnostic and therapeutic strategies.

The development of diagnostic methods, including the assessment of molecular biomarkers, enables earlier detection of the disease, more accurate prognosis assessment, and better prediction of response to treatment. Contemporary colorectal oncology is based on an interdisciplinary approach combining surgery, chemotherapy, radiotherapy, immunotherapy, and targeted therapies, and their selection is increasingly supported by artificial intelligence solutions.

Regardless of the dynamic development of therapeutic methods, the most important element in the fight against colorectal cancer remains regular screening diagnostics. Systematic examinations, such as colonoscopy or fecal occult blood tests, allow for the detection of precancerous lesions and early stages of the disease, which significantly improves the prognosis and treatments’ effectiveness.

Future perspectives in CRC treatment focus on the personalization of therapy, integration of modern diagnostic biomarkers, and optimization of therapeutic algorithms through the use of digital technologies. In the future, it is expected that the combination of classical treatment methods with innovative molecular and immunological strategies, supported by regular population-based screening, will not only improve the prognosis but also enhance the quality of life of patients with colorectal cancer.

## Figures and Tables

**Table 1:** Molecular mechanisms of colorectal cancer’s pathogenesis: comparison of MSI and CIN pathways [17,18,19,22,23,24].

Category	MSI (Microsatellite Instability)	CIN (Chromosomal Instability)
Molecular mechanism	Impairment of the DNA mismatch repair system (MMR—Mismatch Repair)	Numerical and structural changes in chromosomes
Genes involved	*MLH-1*, *PMS-2*, *MSH-6*, *MSH-2*	*APC*, *TP53*, *KRAS*, *SMAD4*
Prevalence in colorectal cancer	12–15%	70–85%
Association with hereditary syndromes	Often associated with Lynch syndrome	Often associated with Lynch syndrome
Typical tumor location	Right (proximal) part of the colon	Distal colon and rectum
Histological appearance	Mucinous carcinoma with marked lymphocytic infiltration	Well or moderately differentiated adenocarcinoma with characteristic glandular architecture

**Table 2:** Characteristics of selected biomarkers used in the diagnosis of colorectal cancer based on [34,36,37].

Biomarker	Clinical Significance
*KRAS* (Kirsten Rat Sarcoma Viral Oncogene Homolog)	An oncogene encoding a GTPase protein (p21) that participates in signal transduction from the cell membrane [36]. Mutations most commonly occur in codons 12 and 13 [37]. The *KRAS* mutation causes permanent activation of the RAS/RAF/MEK/ERK signaling pathway and resistance to anti-EGFR antibody treatment (e.g., cetuximab). *KRAS* mutations occur in up to 40% of patients and have been classified as a predictive factor for a poor response to neoadjuvant treatment in patients with locally advanced rectal cancer [34]. Because mutations in the *KRAS* gene lead to constant activation of the EGFR signaling pathway, epidermal growth factor receptor inhibitors are ineffective [37]. In addition, *KRAS* mutations are more common in tumors located on the left side of the colon and more common in microsatellite-stable colorectal cancer [37].
*BRAF* (v-Raf Murine Sarcoma Viral Oncogene Homolog B1)	A gene encoding a serine–threonine kinase that regulates the MAPK pathway associated with cell proliferation [36]. A mutation in codon 600 (i.e., valine to glutamic acid substitution) occurs in 5–9% of patients with colorectal cancer [37]. The frequency of *BRAF* mutations is higher in women and people over 70 years of age, mainly in the right colon. The *BRAF* mutation is associated with a poorer prognosis, aggressive growth, unfavorable metastasis, and is an indication for testing in patients with stage IV disease to qualify for targeted therapy [36,37].
*TP53* (Tumor Protein p53)	A suppressor gene encoding the cytoplasmic p53 protein, which regulates the cell cycle, apoptosis, aging, and DNA repair. Mutations occur in approximately 60% of CRC patients and are associated with neoplastic transformation (adenoma–carcinoma). However, the detection rate of anti-p53 antibodies in serum has a sensitivity of less than 30%. *TP53* mutations are associated with a poorer prognosis and shorter survival time [36].
*MSI* (Microsatellite Instability)	Occurs in approximately 15% of all colorectal cancers. Caused by a defect in the error correction system during DNA replication—unpaired bases [36,37]. Microsatellite instability is a prognostic marker of a good prognosis and a predictive marker for eligibility for immunotherapy [36]. High microsatellite instability is associated with improved overall survival and a lower rate of disease spread [34]. Knowledge of MSI mutations is crucial in the treatment of colorectal cancer and indicates Lynch syndrome [37].
*APC* (Adenomatous Polyposis Coli)	APC mutations occur in 70–80% of patients with CRC (both in familial adenomatous polyposis and sporadically). The APC protein controls migration, adhesion, transcription, and apoptosis. Methylation of the *APC* promoter does not correlate with survival, but its mutation in combination with high miR-21 expression indicates a poorer prognosis. It may be a biomarker for early detection and a target for targeted therapy [36].
*HER2* (Human Epidermal Growth Factor Receptor 2)	A protein encoded by the erythroblastic oncogene B gene [36]. Increased HER2 expression in patients with colorectal cancer is associated with resistance to anti-EGFR therapy. In addition to its role as a predictive marker for HER2-targeted therapy, it is an indicator of resistance to monoclonal antibodies targeting EGFR [34]. The authors of [37] report that HER2 expression should be assessed in all patients with metastatic colorectal cancer, as it occurs in approximately 2–6% of cases, and its identification is important in terms of therapy and the use of anti-EGFR and anti-HER2 therapies.
*PD-L1* (Programmed Death-Ligand 1)	A surface protein that binds to the PD-1 receptor on T lymphocytes, leading to the inhibition of the immune response, which suppresses the body’s reaction against cancer cells [37]. PD-L1 expression is variable and depends, among other things, on inflammation and the method of sample collection. Some data suggest that the presence of PD-1 on immune cells is associated with a favorable prognosis, and research into new drugs that could expand the use of PD-1/PD-L1-targeted therapies is ongoing [37].

## Data Availability

Not applicable.
